# Dermoid cyst with cerebellar meningoencephalocele at different locations accompanied by posterior fossa abnormalities: case report

**DOI:** 10.1590/1516-3180.2018.0412010418

**Published:** 2018-08-23

**Authors:** Turgut Tursem Tokmak, Ali Koc, Ozgur Karabiyik, Altan Kaya, Mustafa Gureli

**Affiliations:** I MD. Radiologist, Department of Radiology, Kayseri Education and Research Hospital, Kayseri, Turkey.; II MD. Radiologist, Department of Radiology, Kayseri Education and Research Hospital, Kayseri, Turkey.; III MD. Radiologist, Department of Radiology, Kayseri Education and Research Hospital, Kayseri, Turkey.; IV MD. Ear, Nose and Throat Specialist, Department of Ear, Nose and Throat Diseases, Kayseri Education and Research Hospital, Kayseri, Turkey; V MD. Pathologist, Department of Pathology, Kayseri Education and Research Hospital, Kayseri, Turkey.

**Keywords:** Aprosencephaly and cerebellar dysgenesis [supplementary concept], Dermoid cyst, Encephalocele

## Abstract

**CONTEXT:**

Dermoid cysts are well-defined cysts containing sebaceous glands and dermal structures. In the literature, dermoid cysts and associated closure defects have been described in the same locations.

**CASE REPORT:**

In this case, a dermoid cyst was found at the base of the mouth with a coexisting closure defect in the occipital calvarium. Additional abnormalities were also observed, including posterior myeloschisis, right cerebellar dysgenesis, vermian hypogenesis and posterior fusion of the second and third vertebrae. The finding of a dermoid cyst located at the base of the mouth is discussed here, with additional imaging findings.

**CONCLUSION:**

Dermoid cysts in the head and neck region may be accompanied by posterior fossa abnormalities.

## INTRODUCTION

Dermoid cysts are cystic masses that contain different structures such as sebaceous glands, hair follicles and sweat glands within squamous epithelium of ectoderm origin. About 7% of all dermoid cysts are located in the head and neck region. Approximately 11% of these dermoid cysts are found at the base of the mouth, which is the second most common location (the most common location is the lateral eyebrow). Although most of them are benign, slow growing lesions and are common in young adults, it has been reported in the literature that malignant transformation may be found in around 5% of the cases. Coalescence of sebaceous material in the cyst lumen forms a typical “sack of marbles” sign.^1^

Coexistence of dermoid cysts and spinal dysraphism has been documented in many studies. In these studies, dermoid cysts and spinal dysraphism were defined at the same locations. Three cases of a dermoid cyst and coincident encephalocele have been reported in the literature.^1^^,^^2^ To the best of our knowledge, the coexistence of dermoid cyst and midline closure defects/spinal dysraphism at different locations has not previously been mentioned. In the present case report, our aim was to describe an occurrence of a dermoid cyst at the base of the mouth with accompanying occipital cephalocele.

## CASE REPORT

Manuscripts structured as case reports are exempt from approval by our institution’s ethics committee. We received a consent form for reporting on this case.

A 15-year-old girl who was suffering from swelling and pain in the upper neck that had started two months earlier was referred to our hospital. She was evaluated by an ear, nose and throat specialist clinician. On physical examination, there was a painful swelling in the left submandibular region, at the base of the mouth. Deep neck infection was considered as a diagnosis. No abnormality was found through blood tests.

**Figure 1:**
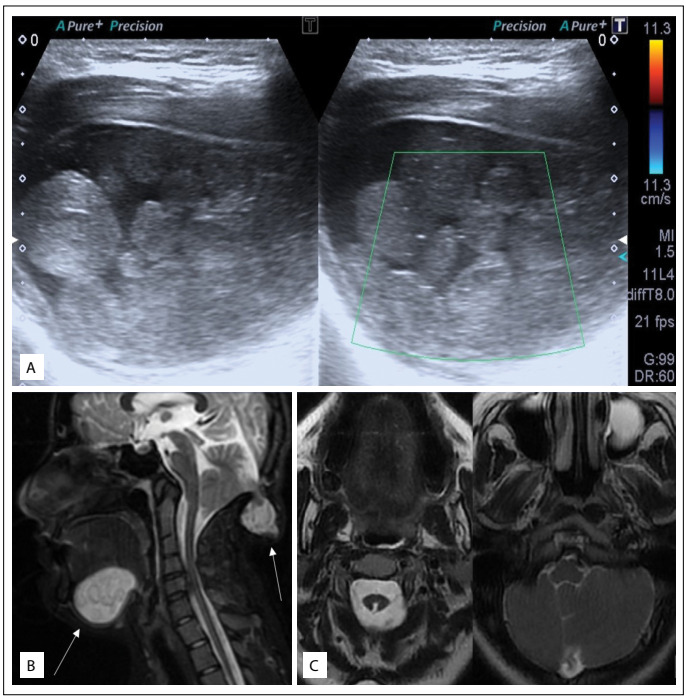
(A) Sonography images showing an oval-shaped, thick-walled cystic lesion. Solid nodular appearance can be seen, without blood flow on color doppler ultrasonography, thus revealing the “sack of marbles” sign. (B) Sagittal T2W Fat Sat image showing hyperintense cystic mass at the base of the mouth, cerebellar encephalomeningocele, tonsillar herniation and C2-C3 vertebral fusion. (C) Axial T2-weighted images demonstrating dysgenesis of the right cerebellum, hypogenesis of the vermis and short-segment posterior myeloschisis at the cervicomedullary junction.

Sonography examination of the neck was performed. Through this, an oval-shaped thick-walled cystic lesion of dimensions 58 mm x 34 mm was detected at the base of the mouth, which extended through the left submandibular region. The lesion appeared to contain dispersed solid nodules that were smaller than 15 mm in diameter, and color doppler sonography showed that there was no blood flow. Thus, a “sack of marbles” sign was revealed (Figure 1). Incidentally, we found that the thyroid echo pattern was heterogeneous, secondary to parenchymal fibrous septa and hypoechoic regions, and was thus consistent with Hashimoto’s thyroiditis.

The laboratory findings were as follows: thyroid-stimulating hormone (TSH) = 0.017 µU/ml (range: 0.27-4.2); free T4 = 2.81 ng/dl (range: 0.93-1.97); and anti-thyroid peroxidase (TPO) = 651 IU/ml (range: 0-40).

Contrast-enhanced magnetic resonance imaging (MRI) was performed for preoperative evaluation of the lesion. MRI showed a thick-walled mass with smooth margins located at the left side of the base of the mouth. The lesion was hypointense on T1-weighted images and hyperintense on T2, and it was composed of small nodules that gave a “sack of marbles” appearance. The rims of the nodules had intermediate signal intensity on T1-weighted images and low signal intensity on T2-weighted images, without signal loss on fat-saturated images. Contrast-enhanced images did not show any enhancement. MRI also showed an occipital bony defect at the midline. There was a cerebellar encephalomeningocele (Figure 1). In addition, partial fusion of the second and third cervical vertebrae was present. Axial brain images demonstrated dysgenesis of the right cerebellar hemisphere, hypogenesis of the vermis, tonsillar herniation and posterior myeloschisis of the cervicomedullary junction (Figure 1).

The medical treatment was planned as if this were a case of hyperthyroidism. Medication was administered before surgery, in order to prevent the complications relating to hyperthyroidism. At surgery, an external transcervical approach was used to enable total excision of the cyst, and there were no complications. No recurrence was detected at an evaluation three months after the surgery. The histopathological diagnosis was reported as a dermoid cyst (Figure 1).

**Figure 2:**
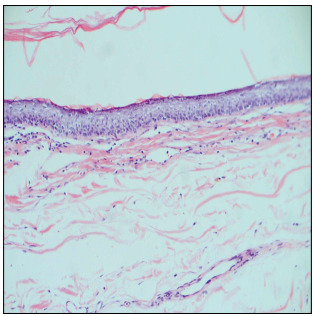
Hematoxylin and eosin staining of the pathological specimen, with original magnification of 40 x. The granular layer containing keratin can be seen on the squamous epithelium of the cyst wall. In addition, lymphocytes can be seen beneath the cyst wall.

## DISCUSSION

Dermoid cysts may be congenital or acquired. The acquired form develops through implantation of epithelial cells into the surrounding tissue, due to trauma or iatrogenic causes. Many congenital dermoid cysts develop at 3-5 weeks of gestation as a result of embryological failure. Epithelial cells are thought to be trapped during the closure of the first and second branchial arches in the formation of dermoid cysts.^1^^,^^3^ True dermoid cysts are lesions that include dermal appendages such as sweat glands, sebaceous glands, hair and hair follicles that are histologically paved with epidermis. A sudden increase in size is observed at the beginning of the puberty, due to the sebaceous glands that they contain.^4^

We used a systematic search in electronic databases (MEDLINE and LILACS) to find articles relating to dermoid cysts and posterior fossa abnormalities (Table 1). Dermoid cysts may be accompanied by midline closure defects, but in the cases that have been reported, cysts and the corresponding closure defects were mostly defined at the same location. Simpson et al.^5^ found coexisting dermoid cysts in the herniated sac in five of their 74 cephalocele cases. They also found concurrent cleft palate (3%), microphthalmia (1%), corneal opacity (1%) and tracheo-esophageal fistula (1%). Posterior fossa anomalies and concomitant occipital encephalomeningocele have been reported in the literature.^5^ In the case reported here, the dermoid cyst was situated at the base of the mouth, while the coexisting closure defect was found in the occipital calvarium, in a different location. In addition, accompanying short-segment posterior myeloschisis, right cerebellar dysgenesis, vermian hypogenesis and C2-3 vertebrae fusion were identified.

**Table 1:** Systematic search of the literature performed in March 2018

Database	Search strategies	Found	Related
MEDLINE (via PubMed)	(“Dermoid Cyst”[Mesh]) AND (“Aprosencephaly and Cerebellar Dysgenesis” [Supplementary Concept]) AND (“Encephalocele”[Mesh])	0	0
LILACS (via BVS)	(“Dermoid Cyst”[Mesh]) AND (“Aprosencephaly and Cerebellar Dysgenesis” [Supplementary Concept]) AND (“Encephalocele”[Mesh])	0	0

Floating fat globules in the cyst can create a characteristic “sack of marbles” appearance. The literature does not provide any knowledge regarding the frequency of the “sack of marbles” sign in dermoid cysts. However, it is known that this sign is indeed pathognomonic for head and neck dermoid cysts.^1^ On sonographic examination, the globules are seen as well-defined hyperechogenic nodule-like structures without blood flow. MRI signals may alter depending on cyst content. On T1W images, these structures are isointense or mildly hyperintense, depending on the lipid content, while they are heterogeneously hyperintense on T2W images. High-lipid content cysts can be seen as dark images through fat-saturated imaging. After administration of contrast medium, mild capsular enhancement may be detected on the cyst wall.^1^ In our case, the dermoid cyst was isointense on T1W images and heterogeneously hyperintense on T2W images, without suppression on fat-saturated images.

In the differential diagnosis for neck dermoid cysts, the following should be considered: thyroglossal duct cyst, inclusion cyst, cystic hygroma, ranula, neoplasms of the sublingual and minor salivary glands, neurofibroma, hemangioma and lymphangioma.

## CONCLUSION

The “sack of marbles” sign in cases of dermoid cysts in the neck is an important and diagnostic finding. Dermoid cysts in the head and neck region may be accompanied by posterior fossa abnormalities. Patients should also be evaluated regarding closure defects.
